# Biological Sulfate Reduction in Deep Subseafloor Sediment of Guaymas Basin

**DOI:** 10.3389/fmicb.2022.845250

**Published:** 2022-03-03

**Authors:** Toshiki Nagakura, Florian Schubert, Dirk Wagner, Jens Kallmeyer, IODP Exp. 385 Shipboard Scientific Party

**Affiliations:** ^1^GFZ German Research Centre for Geosciences, Section 3.7 Geomicrobiology, Potsdam, Germany; ^2^Institute of Geosciences, University of Potsdam, Potsdam, Germany

**Keywords:** sulfate reduction, subsurface life, deep biosphere, thermophiles, Guaymas Basin

## Abstract

Sulfate reduction is the quantitatively most important process to degrade organic matter in anoxic marine sediment and has been studied intensively in a variety of settings. Guaymas Basin, a young marginal ocean basin, offers the unique opportunity to study sulfate reduction in an environment characterized by organic-rich sediment, high sedimentation rates, and high geothermal gradients (100–958°C km^−1^). We measured sulfate reduction rates (SRR) in samples taken during the International Ocean Discovery Program (IODP) Expedition 385 using incubation experiments with radiolabeled ^35^SO_4_^2−^ carried out at *in situ* pressure and temperature. The highest SRR (387 nmol cm^−3^ d^−1^) was recorded in near-surface sediments from Site U1548C, which had the steepest geothermal gradient (958°C km^−1^). At this site, SRR were generally over an order of magnitude higher than at similar depths at other sites (e.g., 387–157 nmol cm^−3^ d^−1^ at 1.9 mbsf from Site U1548C vs. 46–1.0 nmol cm^−3^ d^−1^ at 2.1 mbsf from Site U1552B). Site U1546D is characterized by a sill intrusion, but it had already reached thermal equilibrium and SRR were in the same range as nearby Site U1545C, which is minimally affected by sills. The wide temperature range observed at each drill site suggests major shifts in microbial community composition with very different temperature optima but awaits confirmation by molecular biological analyses. At the transition between the mesophilic and thermophilic range around 40°C–60°C, sulfate-reducing activity appears to be decreased, particularly in more oligotrophic settings, but shows a slight recovery at higher temperatures.

## Introduction

As early as the late 19th century microorganisms were anecdotally reported from surficial deep-sea sediment (Fischer, 1894). In the following decades, several studies mentioned the existence of bacteria in marine sediment (e.g., Rittenberg, 1940; ZoBell, 1952). In their seminal study, Morita and ZoBell (1955) showed the existence of viable microorganisms in abyssal clays from the central Pacific down to several meters depth below the seafloor, but evidence for widespread microbial colonization of deep subseafloor sediment down to hundreds of meters was only presented several decades later (Parkes et al., 1994). While Whelan et al. (1985) showed clear indications of metabolic activity at greater depths based on radiotracer measurements, the presence of an abundant and metabolically active microbial community was only proven at the beginning of the 21st century (Schippers et al., 2005).

In subseafloor sediment, the decrease of microbial abundance with depth is mostly the result of decreasing nutrient availability, whereas increasing temperature and pressure only have a minor effect (Jørgensen, 1982; Middelburg, 1989; Møller et al., 2018; Heuer et al., 2020). However, upon deeper burial, the sediment eventually enters the zone of catagenesis. For sediment from Nankai Trough with mostly terrestrially derived organic matter Horsfield et al. (2006) showed that at temperatures above 60°C, the recalcitrant organic matter such as kerogen is thermally cracked, leading to the production of short-chain organic molecules, which can be utilized *in situ* by microorganisms.

Sulfate-reducing microorganisms (SRM) play a vital role in biogeochemical cycling of sulfur and carbon and therefore are key players in the regulation of global climate (Pester et al., 2012). Sulfate is reduced to hydrogen sulfide which, particularly in marine sediment, further reacts with other ions and organic matter and forms a variety of reduced organic and inorganic sulfur species. The sum of reduced inorganic sulfur species (ΣH_2_S + FeS + FeS_2_ + S^0^) is also called Total Reduced Inorganic Sulfur (TRIS; Howarth, 1979; Howarth and Teal, 1979; Howarth and Giblin, 1983).

Sulfate concentrations are high near the sediment–water interface due to downward diffusion of sulfate from the overlying water column. With increasing depth sulfate concentration decreases as the rate of sulfate consumption exceeds the downward flux. There are two pathways of microbial sulfate reduction:

1. Organoclastic sulfate reduction: 2CH_2_O + SO_4_^2−^ = 2HCO_3_^−^ + H_2_S
2. Methanotrophic sulfate reduction: CH_4_ + SO_4_^2−^ = HCO_3_^−^ + HS^−^ + H_2_O

Sulfate-reducing microorganisms can metabolize various carbon substrates, such as fatty acids (Widdel, 1980; Widdel and Pfennig, 1981), aromatic compounds (Widdel, 1980), sugars (Sass et al., 2002), amino acids (Baena et al., 1998), and, in a consortium with archaea, methane (Boetius et al., 2000). The selection of a specific metabolic pathway is based on the availability of substrates. In near-surface sediments, volatile fatty acids (VFAs) are produced by fermentation of macromolecular organic matter. Due to preferential degradation of easily degradable organic matter, the reactivity of the remaining bulk organic matter decreases with increasing burial depth (Middelburg, 1989), leading to lower production rates of VFAs and hence lower sulfate reduction rates (SRR; Hoehler and Jørgensen, 2013).

Methanogenesis is the final step in the degradation of organic matter, once all other electron acceptors with a higher energy yield have been depleted (Froelich et al., 1979). The upward diffusing methane eventually reaches depths where sulfate is still available, the so-called sulfate–methane transition zone (SMTZ) in which sulfate and methane coexist. In the SMTZ, methanotrophic sulfate reduction (i.e., anaerobic oxidation of methane, AOM) takes place. Although the concentration of sulfate is extremely low below the SMTZ, sulfate still exists due to the downwards diffusion of sulfide and re-oxidation to sulfate by reactive iron species. Thus, biological sulfate reduction can occur slowly below the SMTZ (Holmkvist et al., 2011).

With recent improvements in drilling techniques, exploration of the subseafloor biosphere ventured into great depths with correspondingly decreased microbial abundance and metabolic activity (Hoehler and Jørgensen, 2013; Ciobanu et al., 2014; Heuer et al., 2020). The low turnover rates encountered in the deep subsurface require highly sensitive quantification techniques. Since the first measurement of SRR with ^35^S-radiotracer by Sorokin (1962), the method was improved several times to make it more user friendly, accurate, and sensitive (Howarth and Giblin, 1983; Canfield et al., 1986; Fossing and Jørgensen, 1989; Hsieh and Yang, 1989; Kallmeyer et al., 2004; Røy et al., 2014).

Microbial sulfate reduction occurs over almost the entire known temperature range of life, including the hyperthermophilic range > 80°C (Weber and Jørgensen, 2002). Even at temperatures exceeding 100°C sulfate reduction has been detected (Jørgensen et al., 1992; Elsgaard et al., 1994). High SRR were recorded near hydrothermal vents where a high flux of readily bioavailable low molecular weight carbon compounds fuel high per-cell activity of the SRM, which is necessary to maintain cellular integrity under extreme temperature conditions (Weber and Jørgensen, 2002).

**Figure 1:**
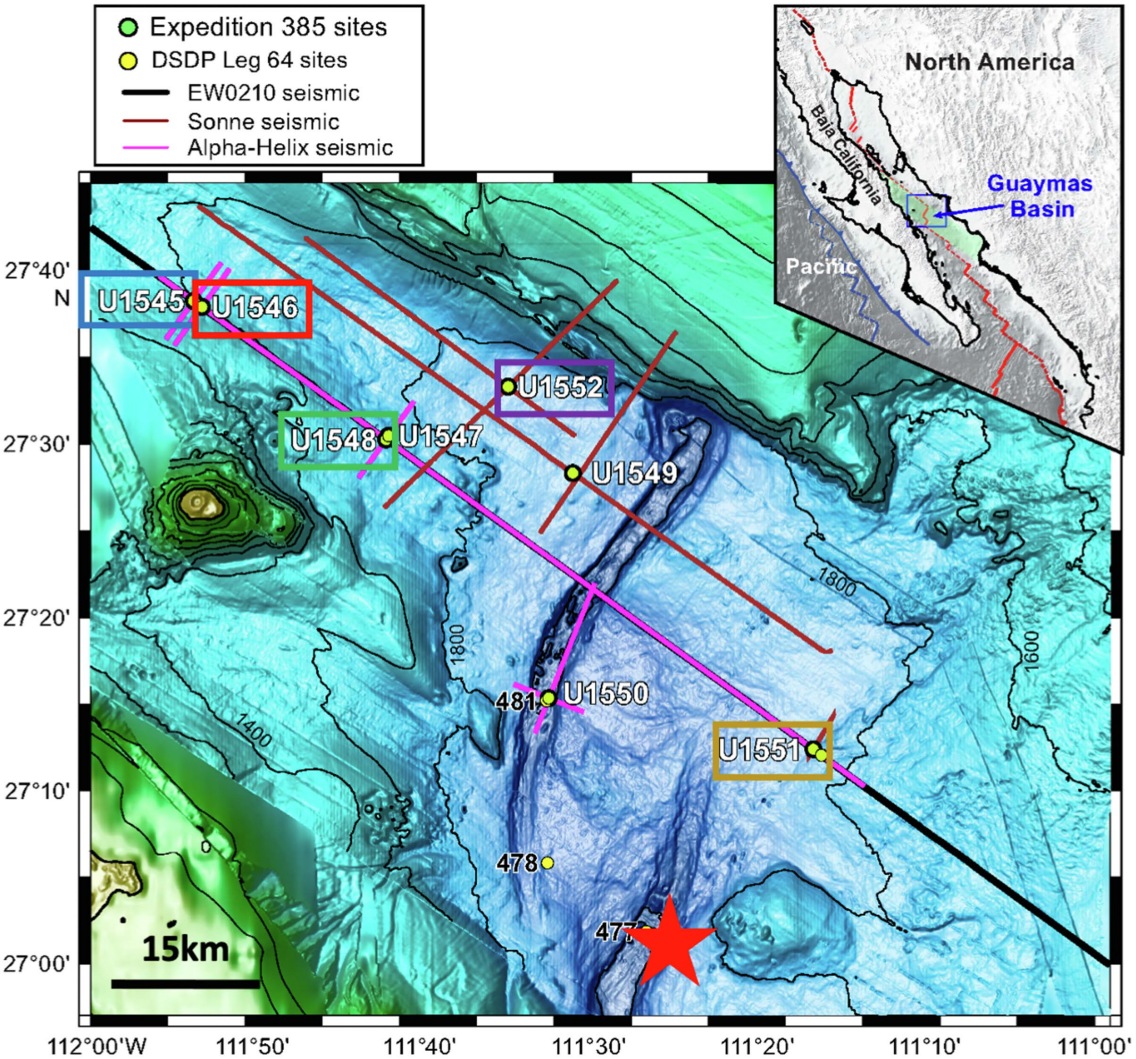
Sampling sites in Guaymas Basin. Image adapted from Teske et al. (2020). Colored boxed indicate sampling sites where SRR could be measured. The color scheme is used for data plotted in Figures 2–4. The red line in the insert map is the boundary between the North American and Pacific tectonic plates. The red star in the map indicates the sampling site of Elsgaard et al. (1994).

Guaymas Basin, located in the Gulf of California, Mexico, is a young marginal ocean basin of approximately 1,100 km long and 200 km wide that connects to the eastern Pacific Ocean (Figure 1). The high sedimentation rates of >1 mm year^−1^ result in the mainly biociliceous fine-grained sediment containing abundant organic matter of both marine and terrestrial origin (van Andel, 1964; Curray et al., 1979; Teske et al., 2020). Since thick sediments are covering the active seafloor spreading there are hydrothermal vents on the seafloor and frequent basaltic sill intrusions into the sediment. Geothermal gradients in the subsurface of Guaymas Basin are reaching up to 1,000°C km^−1^ (Teske et al., 2020). Due to this peculiar geological setting, the bioavailability of organic substrates is expected to not follow the expected decrease as in non-hydrothermal sediments. One of the main objectives of the International Ocean Discovery Program (IODP) Expedition 385 (Guaymas Basin Tectonics and Biosphere) was the investigation of microbial communities and activity in the deep subseafloor biosphere of Guaymas Basin. Detailed drill site descriptions are given by Teske et al. (2020, 2021) (Table 1). Sites U1545C and U1546D have a similar stratigraphy since the distance between these two sites is only about 1.1 km. However, while the sediment at Site U1545C reveals an undisturbed sedimentary profile, the sediment in Site U1546D was intruded by a sill around 350–430 m below seafloor (mbsf), thus these two sites offer a chance to study the impact of sill intrusion on sedimentary biogeochemical cycling.

**Table 1:** Summary of drill sites of International Ocean Discovery Program (IODP) Exp. 385 (Teske et al., 2020, 2021) from which samples were used for this study.

Site and Hole	Latitude and Longitude	Temperature gradient (°C km^−1^)	Seafloor depth (mbsl)	Number and depth range of samples	Site description
U1545C	27°38.2420’N 111°53.3290’W	225	1,595	21, 2.0–325.8 mbsf	About 52 km northwest of the western axial graben of the northern Guaymas Basin spreading segment, sediment strata are not disturbed by the intruding sill, 1.1 km distance to Site U1546D, mainly diatom ooze and clay.
U1546D	27°37.8943’N 111°52.7812’W	221	1,586	18, 2.1–292.4 mbsf	About 51 km northwest of the western axial graben, disturbed sedimentary layer due to the sill intrusion at approx. 350–430 mbsf, mainly diatom ooze and diatom clay.
U1548C	27°30.2698’N 111°40.8476’W	958	1,737	7, 1.9–59.8 mbsf	About 27 km northwest of the western axial graben, located at the outside of the active hydrothermal ringvent, mainly diatom ooze.
U1551B	27°12.3832’N 111°13.1841’W	100	1,844	5, 1.4–35.6 mbsf	About 29 km southeast of the western axial graben, the only sampling site from the southeastern region of Guaymas Basin among the eight sampling sites, and predominantly terrestrial silt and sand.
U1552B	27°33.2885’N 111°32.9640’W	262	1,841	5, 2.1–48.0 mbsf	About 20 km northwest of the western axial graben, cold seep seafloor, gas hydrate rich site, diatom/silty clay, and sandy silt.

*mbsl stands for meter below sea level and mbsf stands for meter below seafloor. See Supplementary Table S1 for a list of all SRR data*.

Sites U1547B and U1548C are located inside and outside of a ringvent (circular hydrothermal mound), respectively. Around these sampling sites, sills intruded into the sediment at relatively shallow depths; *ca*. 150 mbsf at Site U1547B and *ca*. 65 mbsf at Site U1548C. This hydrothermally active area was chosen to study the effects of extremely steep geothermal temperature gradients (958°C km^−1^ at Site U1548C) on biological processes.

Site U1551B is the only site from the southeast region of Guaymas Basin. Sediment at this site is of predominantly terrestrial region. Site U1552B is located close to cold methane seeps and gas hydrates were recovered.

Although SRR were measured previously in Guaymas Basin, the studies focused only on the top 40 cm sediment (Jørgensen et al., 1992; Elsgaard et al., 1994; Weber and Jørgensen, 2002), thus the rates and distribution of microbial sulfate reduction in the deep subseafloor and thermal effect on microbial sulfate reduction are still unclear. We investigated microbial sulfate reduction down to 326 mbsf in subsurface sediment of Guaymas Basin, using ^35^S radiotracer incubations in the laboratory at approximate *in situ* temperature and pressure.

## Materials and Methods

### Sampling

Core samples were collected from eight drill sites during IODP Exp. 385 (Guaymas Basin Tectonics and Biosphere) from 16 September 2019 to 16 November 2019 (Teske et al., 2020). Figure 1 provides an overview of the sampling area of Exp. 385; detailed information about the sampling sites is given in Table 1. An advanced piston corer (APC)^1^ can recover soft sediments without disturbing and the half-length APC (HLAPC)^2^ is employed for firm sediments that are not suitable to use APC. Both coring systems are dedicated to recovering high-quality samples with minimal contamination from drilling fluid, which is a prerequisite for any geomicrobiological or biogeochemical analyses (Kallmeyer, 2017; IODP Tech. Note). Concentrations of pore water methane and sulfate as well as TOC were carried out on board according to established IODP protocols.^3^ Data were taken from the IODP database.^4^

### Subsampling, Storage, and Sample Processing

Immediately after retrieval of the core, whole round core (WRC) segments were cut off and capped. The WRC was then brought into the ship’s lab, put into gas-tight aluminum foil bags flushed with N_2_ gas, and stored at *ca*. 4°C shipboard, in transit, and until use in the home lab. Unfortunately, due to a technical malfunction on board the drill ship, some samples were stored in an oxic atmosphere for several weeks. Samples from Site U1547B were compromised by the extended exposure to oxygen and no reliable SRR measurements could be conducted. Samples from sites U1549B and U1550B also suffered from the exposure to oxygen albeit for much shorter duration. SRR measurement of samples from sites U1549B and U1550B was partially successful but the quality of the data remained inconclusive. The SRR data for these sites, therefore, were also discarded. For this study, we used only SRR measurements from uncompromised samples from sites U1545C, U1546D, U1548C, U1551B, and U1552B.

### Preparation of Media

The seawater media for the SRR measurement were prepared as follows, based on Morono et al. (2017): 0.2 g KH_2_PO_4_, 0.25 g NH_4_Cl, 25 g NaCl, 0.5 g MgCl_2_ × 6H_2_O, 0.5 g KCl, and 0.15 g CaCl_2_ × 2H_2_O, were mixed with 1 L of ultrapure water (UPW). About 3 ml of 0.1% resazurin was added to the media and autoclaved. About 5 ml of Na_2_S solution (0.12 g Na_2_S in 10 ml UPW) and 5 ml of NaHCO_3_ solution (0.84 g NaHCO_3_ in 10 ml UPW) were added to the media after autoclaving. The media were bubbled with N_2_/CO_2_ gas for at least 2 h. After cooling, it was stored in pre-combusted crimp bottles with N_2_ gas until use within a few days. For all samples from sites U1545C and U1546D, 0.71 g Na_2_SO_4_ was also added to set the sulfate concentration to 5 mM. For samples from sites U1548C, U1551B, and U1552B, we approximated the *in situ* porewater sulfate concentration by adding a separately prepared sterile 1 M Na_2_SO_4_ stock solution to those samples.

### Control Samples

Killed controls (KC) and media controls (MC) were also prepared and incubated, alongside with the regular samples. For KC, 20% of zinc acetate was added to the sample instead of medium to stop all microbial activity. MC consisted of only the sterile medium and no sediment.

### Incubation of Samples

**Figure 2:**
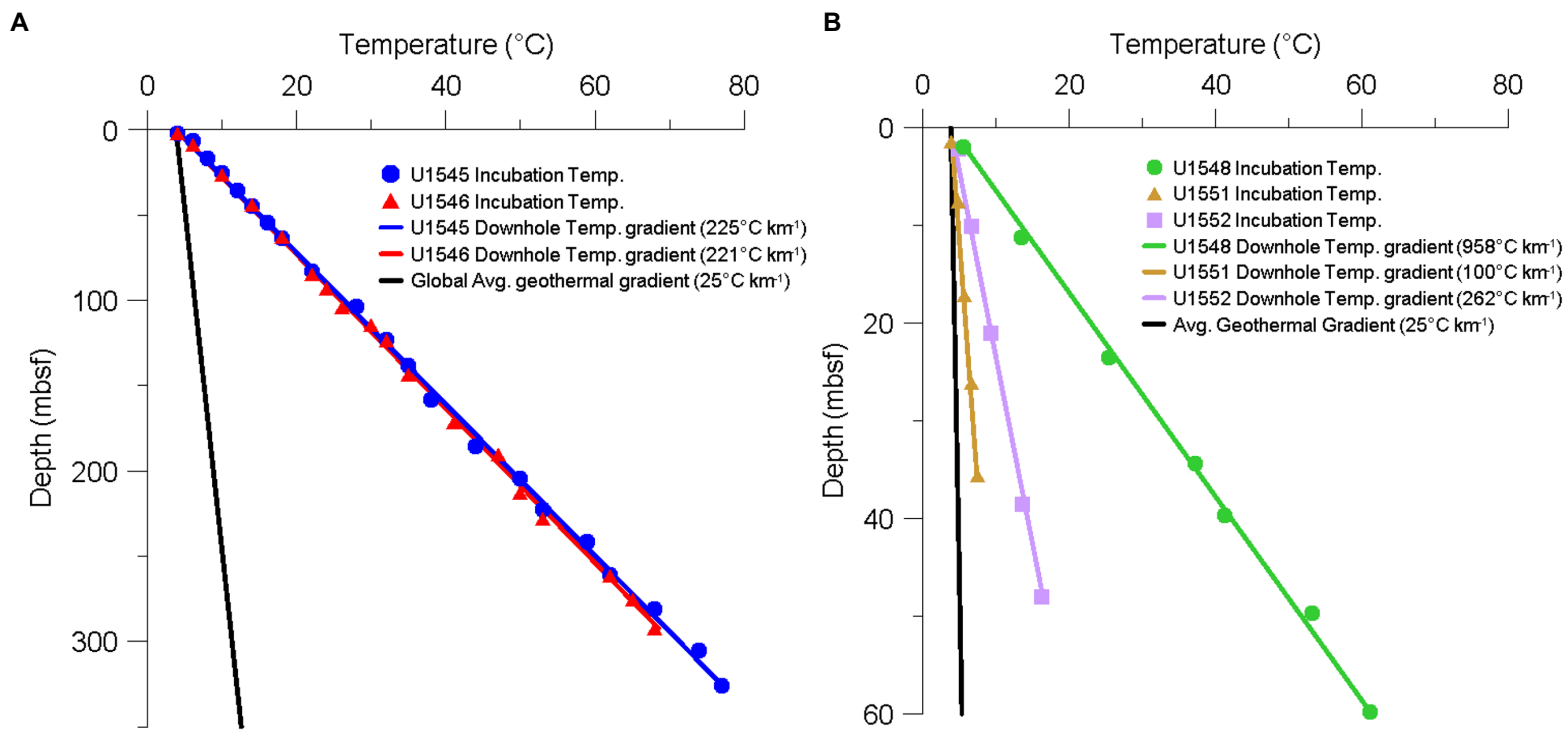
Temperature gradients of the different sampling sites of IODP Exp. 385. **(A)** Downhole temperature gradients of sites U1545 (blue line) and U1546 (red line) measured *in situ*. Blue circles and red triangles show sampling depth and incubation temperature of each sample from sites U1545C and U1546D, respectively. **(B)** Downhole temperature gradients of sites U1548 (green line), U1551 (brown line), and U1552 (purple line) measured *in situ*. Green circles, brown triangles, and purple squares show sampling depth and incubation temperature of each sample from sites U1548C, U1551B, and U1552B, respectively. The black lines in both figures show the average global geothermal gradient of 25°C km^−1^. Note the different depth scales on the two graphs.

All experiments were carried out at the GFZ German Research Centre for Geosciences, Potsdam, Germany. Inside an anaerobic glove box, 10 g of sample were weighed and put into pre-combusted glass vials. Medium was added and the vial was closed with a black butyl rubber stopper and crimped. To allow for more flexibility of the stopper and improved pressure transmission and hence avoiding breakage of the glass vial upon pressurization or depressurization, we cut off the bottom *ca*. 5 mm of the stopper. Particularly, at low temperatures, we found that the flexibility of the stoppers was severely reduced, leading to broken vials. We therefore stuck a 3 ml syringe with a cut-off plunger, containing 0.5 ml of medium into each vial (Nauhaus et al., 2002). The syringe can accommodate volume changes irrespective of incubation temperature. The samples were pre-incubated at 4°C overnight at ambient pressure. Then, 5 MBq of ^35^SO_4_^2−^ were injected into each sample, KC, and MC. Afterward, all vials were put into high-pressure cylinders, which were then placed in a high-pressure thermal gradient block (HPTGB) similar to the design of Kallmeyer et al. (2003). The HPTGB system consists of a thermally insulated aluminum block of 150 cm × 20 cm × 20 cm with three rows of 15 slots each, each slot is holding a stainless-steel high-pressure vessel. Temperature can be adjusted at the two ends of the block, allowing for a thermal gradient of ∆T_max_ 107°C, and minimum and maximum temperatures of −5 and 155°C, respectively. Pressure is created by high-pressure liquid chromatography pumps (Sykam, Fürstenfeldbruck, Germany), running at a constant flow rate against a back-pressure regulator (Swagelok, Germany) to prevent pressure fluctuations during thermal equilibration of the samples. The cylinders were pressurized to an approximate *in situ* pressure of 25 MPa and incubation ran for 10 days. Figure 2 shows the incubation temperature of each sample, together with the downhole temperature gradient. All samples were incubated within ±2°C of their respective *in situ* temperature. Incubation was terminated by depressurization of the high-pressure cylinder, followed by removal of the crimp vial from the pressure cylinder, opening of the vial, and quantitative transfer of its contents into a 50 ml centrifuge tube preloaded with 5 ml of 20% zinc acetate solution to terminate microbial activity. Remaining sediment pieces were flushed out with 10 ml of 20% zinc acetate solution to transfer all of the incubated sediment into the centrifuge tube. All samples were stored at −20°C until analysis.

### Distillation of the Samples

We used a slightly modified version of the cold chromium distillation of Kallmeyer et al. (2004) to liberate the reduced sulfur species from the sediment. After thawing the samples, they were centrifuged for 10 min at 2,500 × *g*. For quantification of total radioactivity (a_TOT_), we mixed 50 μl of the supernatant with 4 ml of scintillation cocktail (Rotiszint® eco plus LSC-Universalcocktail, Carl Roth, Karlsruhe, Germany). The rest of the supernatant was carefully decanted off. The sediment pellet was then quantitatively transferred to the distillation flask and mixed with 15 ml N, N-dimethylformamide (DMF), technical grade. To ensure complete mixing of the sediment with the chemicals, a magnetic stir bar was added to the reaction flask. The flask was connected to a constant stream of nitrogen gas (approximately 5–10 bubbles per second) in order to maintain strictly anaerobic conditions during the distillation. After 10 min of bubbling, the DMF-sediment suspension with N_2_ gas, 8 ml of 6 M hydrochloric acid (HCl), and 15 ml of 1 M chromium (II) chloride solution were added to the flask *via* a reagent port. The chemicals will convert all reduced sulfur species (TRIS) to H_2_S, which is driven out of solution by the stream of nitrogen gas. The produced gas is then led from the flask through a Poly-Ether-Ether-Ketone (PEEK) tube to the citrate trap filled with 7 ml of a buffered citric acid solution (19.3 g citric acid, 4 g NaOH in 1 l H_2_O, and pH 4) to trap any aerosols potentially containing unreacted ^35^SO_4_^2−^ radiotracer but let all H_2_S pass. Finally, the gas is led to a trap containing 7 ml of 5% (w/v) zinc acetate. To prevent overflowing of the zinc acetate trap, a few drops of silicon-based antifoam are added. After 2 h of distillation, the contents of the zinc acetate trap, containing the produced H_2_^35^S are quantitatively transferred into a 20 ml plastic scintillation vial and mixed with 8 ml scintillation cocktail for quantification of radioactivity by scintillation counting. Each round of distillations also included one distillation blank (DB) containing just a few drops of non-radioactive zinc sulfide carrier and the distillation chemicals mentioned above. The DB was used to detect potential carry-over between distillation runs and therefore tracks the cleanliness of the distillation equipment. Counter blanks (CB) containing just 5% zinc acetate and scintillation cocktail in the same ratios as the samples, are added to each run of the scintillation counter. Counter blanks are required to quantify the background activity, i.e., all signals not associated with radioactivity. We use a HIDEX 600 SL Liquid Scintillation Counter (HIDEX Oy, Finland) with Guard Scintillator. Before the vials are put into the counter, they are vortexed, and the surface of the vial is wiped with 70% ethanol in order to remove any potential contamination on the vial’s surface. Since the samples recovered during the expedition were incubated as slurries with additional medium, the total incubation conditions deviate considerably from *in situ* conditions. The measured rates should therefore be considered “potential” SRR (pSRR).

### Calculation of Sulfate Reduction Rates and Minimum Quantification Limit and Minimum Detection Limit of SRR

The formula of Jørgensen (1978) for calculation of SRR based on radioisotope incubations was designed for whole core incubations, not slurries with added medium. We therefore use a revised version:


$$\begin{array}{l} \mathrm{SRR}=\left({\left[{\mathrm{SO}}_{4}{}^{2-}\right]}_{\mathrm{PW}}\times {\text{P}}_{\mathrm{SED}}\times {\text{V}}_{\mathrm{SED}}+{\left[{\mathrm{SO}}_{4}{}^{2-}\right]}_{\mathrm{MEDIA}}\times {\text{V}}_{\mathrm{MEDIA}}\right)\\ \;\;\;\;\;\;\;\;\;\;\;\;\;\;\;\;\;\;/{\text{V}}_{\mathrm{SED}}\times a_{\mathrm{TRIS}}/a_{\mathrm{TOT}}\times 1/\text{t}\times 1.06\times 1,000,000 \end{array}$$

where SRR is calculated in pmol cm^−3^ d^−1^, [SO_4_^2−^]_PW_ is the sulfate concentration (μmol cm^−3^) of the sediment porewater, *P*_SED_ is the porosity of the sediment, *V*_SED_ is the volume of the sediment (cm^3^), [SO_4_^2−^]_MEDIA_ is the sulfate concentration (μmol cm^−3^) of the media, *V*_MEDIA_ is the volume of the media (cm^3^), *a*_TRIS_ is the radioactivity found in the TRIS fraction, *a*_TOT_ is total radioactivity used, *t* is incubation time (d), 1.06 is the correction factor for the expected isotopic fractionation (Jørgensen, 1978), and 1,000,000 is the factor to convert to pmol (Kallmeyer et al., 2004).

Any analytical method, in our case liquid scintillation counting (LSC), has an inherent background and uncertainty. The background (or blank) is a signal to which several factors contribute, but all of them are neither associated with radioactive decay nor with biological turnover of tracer. Ultra-low turnover rates require work at the absolute limit of detection, which in turn requires a strict separation of the background signal and “real” turnover. For assessment of turnover, we use the definitions of minimum quantification limit (MQL) and minimum detection limit (MDL) of Kaiser (1970):

MDL = mean value of blanks
MQL = MDL + *k* × σ*b*

Where *k* is the coverage factor associated with the level of confidence, *b* is the blank and σ*b* is the SD of the background. Both mean value and SD were calculated for all control samples (KC and MC) and blanks (DB and CB), which were processed and measured together with the samples. A coverage factor of *k* = 3 is chosen to represent a level of confidence of 95% as recommended by Kaiser (1970) and Currie (1968) for one-tailed probabilities, i.e., false positives will only occur above MDL and not below.

The only parameter where the MQL and MDL become relevant is *a*_TRIS_, due to the extremely low microbial activity in our samples. As we were using 5 MBq per sample, *a*_TOT_ is always well above the detection limit. Values of *a*_TOT_ < MDL are discarded, for values MDL < *a*_TOT_ < MQL and *a*_TOT_ > MQL, we calculate a SRR. SRR calculated from values *a*_TOT_ > MQL represent actual turnover measurements and exclude false positives with a level of confidence of 95%, whereas SRR calculated from values MDL < *a*_TOT_ < MQL are rates that could be detected but cannot be distinguished from the inherent background of LSC with a sufficient level of confidence. Those rates are plotted as well, but with different symbols. The calculated SRR depends on sulfate concentration and the *a*_TRIS_/*a*_TOT_ ratio, therefore the MQL is different for each sample.

## Results

### Sulfate Reduction Rates at Sites U1545C and U1546D

**Figure 3:**
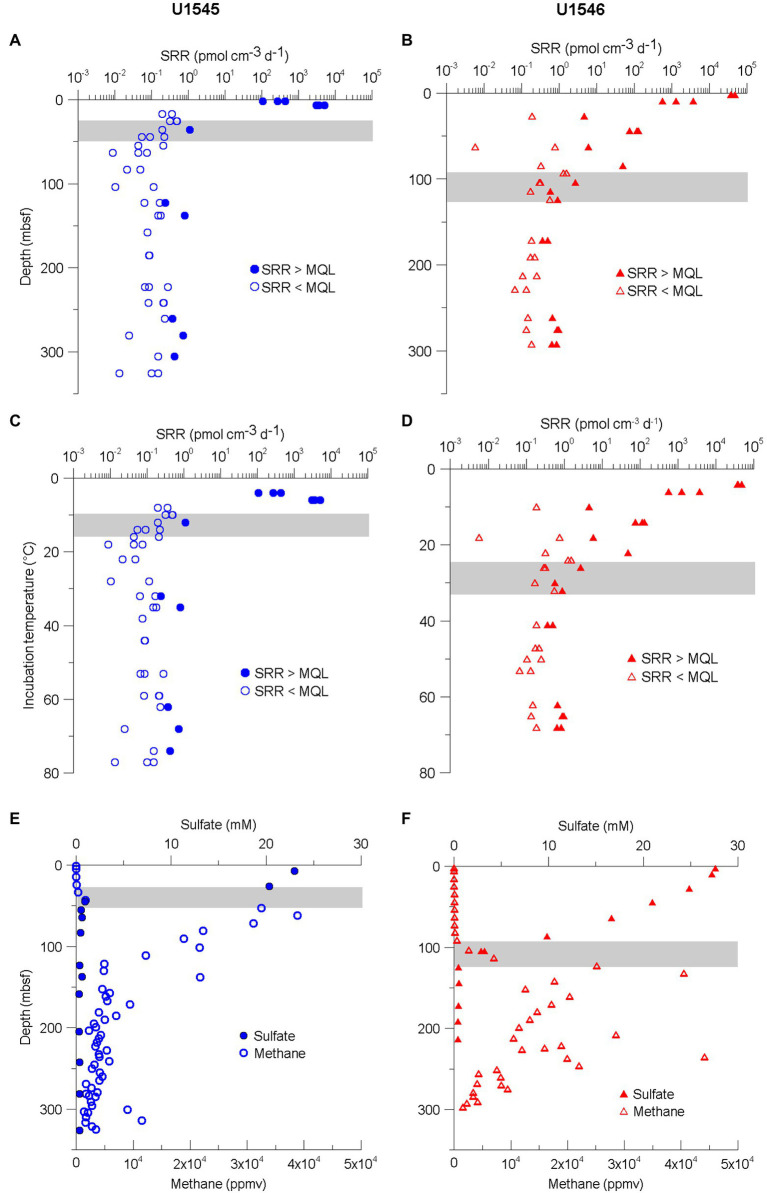
**(A,B)** Sulfate reduction rates (SRR) for sites U1545C (blue circles) and U1546D (red triangles) plotted against sampled depth. Closed symbols indicate measurements SRR > MQL and open symbols MDL < SRR < MQL. See the definitions of MQL and MDL in the “Materials and Methods” section. **(C,D)** SRR for same sites plotted against incubation temperature. **(E,F)** Concentrations of sulfate and methane. The data were obtained from the Laboratory Information Management System (LIMS) report. Gray bar indicates Sulfate–Methane Transition Zone (SMTZ).

The SRR for sites U1545C and U1546D are shown in Figures 3A–D. For sites U1545C and U1546D, the original objective in terms of microbiology was to elucidate the impact of sill intrusion on microbial activity at sites with similar sediment composition, TOC content (scattering between 1 and 2.5%) and stratigraphy but different sill intrusion histories. During the expedition downhole, temperature measurements indicated that the sill located at Site U1546D had reached thermal equilibrium with the surrounding sediment (Teske et al., 2021), so there is no local heating effect at present. Both sites have relatively high SRR near the seafloor, with maxima of 5.1 and 48.5 nmol cm^−3^ d^−1^ and with minima above MQL of 0.2 and 0.4 pmol cm^−3^ d^−1^ for sites U1545C and U1546D, respectively (Figures 3A,B). Sulfate-reducing activity was detectable down to about 300 mbsf at both sites. From the sediment surface down to about 100 mbsf, SRR at Site U1546D are higher than at Site U1545C. From *ca*. 10 mbsf, SRR drop by several orders of magnitude with depth at Site U1545C, while at Site U1546D SRR decrease more gradually. Below 10 mbsf at Site U1545C and 100 mbsf at Site U1546D, we could detect SRR around 300 fmol cm^−3^ d^−1^ and many samples fell below our minimum detection limit. The depths at which SRR fall to near background rates coincide with the respective depths of the SMTZ, which are located around 40 mbsf at Site U1545C and around 110 mbsf at Site U1546D (Figures 3E,F). SRM show metabolic activity over a temperature range from 4 to 74°C (Figures 3C,D), thus the SRM inhabiting the subsurface of Guaymas Basin range from psychrophiles to thermophiles. Interestingly, at both sites, there is a depth interval in which all SRR measurements fell below MQL. At Site U1545C, the SRR < MQL interval reaches from 158.1 mbsf (38°C) to 241.6 mbsf (59°C), whereas at Site U1546D the interval is between 190.7 mbsf (47°C) and 228.4 mbsf (53°C). Above 60°C, in the thermophilic range, SRR start to increase again at both sites (Figures 3C,D).

### Sulfate Reduction Rates at Sites U1548C, U1551B, and U1552B

**Figure 4:**
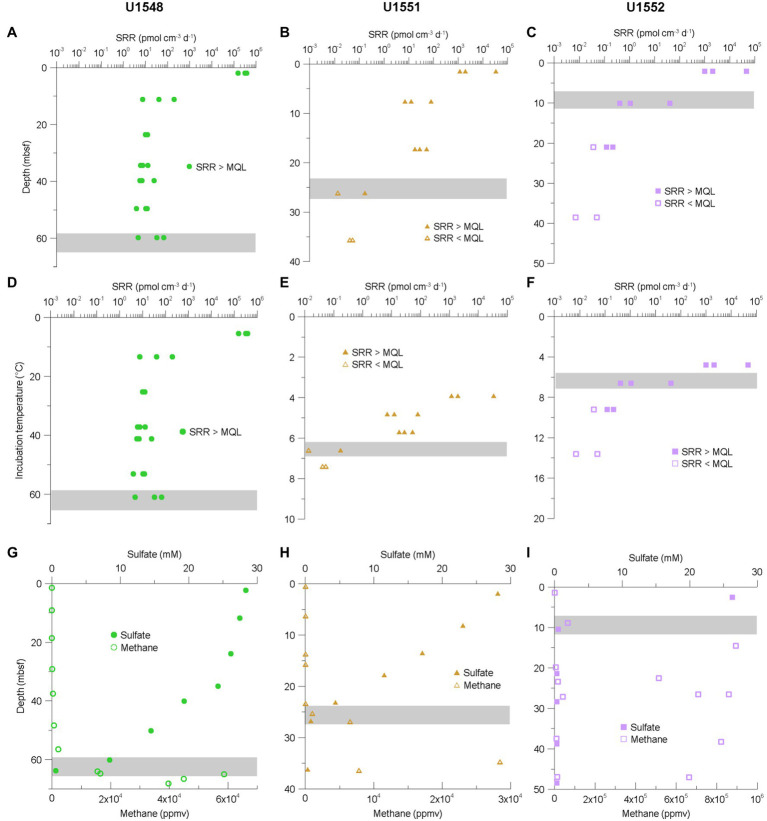
**(A–C)** SRR for sites U1548C (green circles), U1551B (brown triangles), and U1552B (purple squares) plotted against sampled depth. **(D–F)** SRR for same sites plotted against incubation temperature. **(G–I)** Concentrations of sulfate and methane. The data were obtained from the Laboratory Information Management System (LIMS) report. Gray bar indicates Sulfate–Methane Transition Zone (SMTZ). Note the different scales on the graphs.

The highest SRR value at sites U1548C, U1551B, and U1552B, 387 nmol cm^−3^ d^−1^, was measured in a sample from 1.9 mbsf at Site U1548C (Figure 4A), which exhibits the highest geothermal gradient (958°C km^−1^) among the eight expedition sites. The deepest sample (59.8 mbsf) at this site was located 10 m above a sill and revealed SRR of 4.8–68 pmol cm^−3^ d^−1^ at 61°C (Figures 4A,D). All SRR measurements from Site U1548C were above the MQL.

For sites U1551B and U1552B, SRR in near-surface samples are one to two orders of magnitude lower than that at Site U1548C, revealing maximum values of 34.2 and 46.1 nmol cm^−3^ d^−1^, respectively (Figures 4B,C). The temperature ranges of samples retrieved from these two sites are between 3.9 and 7.4°C at Site U1551B and between 4.8 and 16.2°C at Site U1552B but SRR above the MQL could only be detected in samples with temperatures of up to 6.6°C (0.2 pmol cm^−3^ d^−1^) and 9.2°C (0.1 pmol cm^−3^ d^−1^; Figures 4E,F).

Owing to technical problems with our scintillation counter, we were not able to use the blank measurements (KC, MC, DB, and CB) from the U1548C samples. In order to calculate the MQL, which is crucially important to assess the SRR data, we used blank measurements (KC, MC, DB, and CB) from the Site U1547B samples.

A list of all SRR measurements is provided in the Supplementary Table S1.

## Discussion

### Sulfate-Reducing Activity in Deep Subsurface Sediment of Guaymas Basin

**Figure 5:**
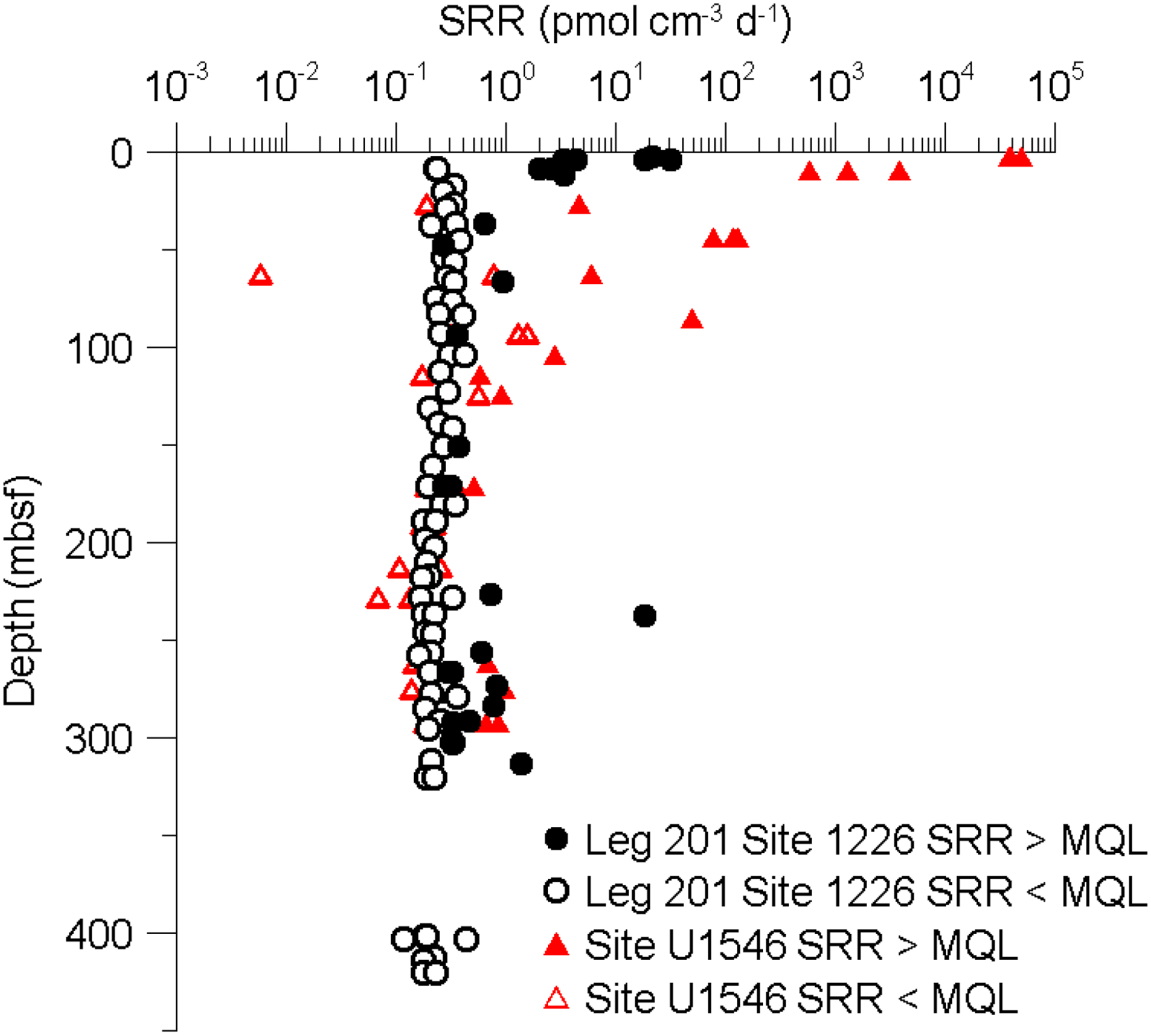
Comparison of SRR in subsurface sediments from the tropical eastern Pacific Ocean (ODP Leg 201, Site 1,226) and Site U1546D.

We quantified SRR in subsurface sediments from several sites at Guaymas Basin, covering a wide range of geothermal gradients and depositional settings. One aspect of the IODP Exp. 385 was investigating the microbial community in this unique subseafloor environment. Ocean Drilling Program (ODP) Leg 201 was the first ocean drilling project dedicated to the study of subseafloor life. Several sites in the eastern Pacific Ocean and Peru margin were drilled to gain knowledge about the environmental conditions in deep marine subsurface sediments (D'Hondt et al., 2004; Jørgensen et al., 2006). Compared to ODP Leg 201 Site 1226 in the tropical Pacific Ocean (Parkes et al., 2005), SRR are generally higher in Guaymas Basin, particularly, near the seafloor. At depths of tens and hundreds meters below seafloor, SRR are in about the same range (Figure 5). Various reasons contribute to higher turnover rates in Guaymas Basin: firstly, Guaymas Basin sediment is characterized by a generally higher content of organic matter than sediments from open ocean sites (near seafloor samples at sites U1545C and U1546D have >3.5 wt% TOC, whereas at Site 1226 TOC concentrations are <1%). Secondly, the steep geothermal gradients lead to increased availability of organic substrates generated through thermal cracking of otherwise unavailable macromolecular organic matter (Horsfield et al., 2006) at comparatively shallow depths. A third potential factor appears to be local heating around sills to further elevate microbial activity, at least temporarily.

Compared to the data of Elsgaard et al. (1994), who quantified SRR in the upper 40 cm of hydrothermal sediment from the southern trough of Guaymas Basin (Figure 1), near-seafloor samples from our study revealed similar or higher SRR. The higher SRR could be a result of incubation of our samples at approximate *in situ* pressure (25 MPa), whereas Elsgaard et al. (1994) carried out all experiments at atmospheric pressure. Using slurries of surface sediment from Guaymas Basin, Kallmeyer et al. (2003) showed that SRR increased by almost an order of magnitude when the incubation was carried out at *in situ* pressure (*ca*. 25 MPa) instead of atmospheric pressure. Interestingly, when applying even higher pressure (45 MPa) SRR increased even further, but the increase in SRR when raising the pressure from 25 to 45 MPa was not as pronounced as for the pressure increase from 0.1 to 25 MPa.

Sulfate concentration in the media used for all samples from sites U1545C and U1546D was set to 5 mM, which is lower than *in situ* porewater sulfate concentrations of near-surface samples and higher for the deep samples. Although the sulfate concentration of the deep samples was increased, SRR of most of the deep samples were below MQL, which we interpret as either electron donor (organic substrate) limitation or a generally very small or metabolically slow SRM community that could not adapt to elevated sulfate levels within the 10 days incubation time.

Abiological sulfate reduction, also termed thermochemical sulfate reduction (TSR), is assumed to occur only at temperatures well over 100°C (Jørgensen et al., 1992; Machel, 2001) and turnover rates are much slower than biological sulfate reduction, we thus assume that SRR measured in our study are exclusively caused by microbial activity.

### Effect of Sill Intrusion on the Microbial Sulfate-Reducing Activity

Although a thick sill was predicted from seismic data and observed at Site U1546D, the expected higher geothermal gradient was not observed, with current geothermal gradients at sites U1545C and U1546D being almost identical. This indicates that the sill at Site U1546D has reached thermal equilibrium with its surrounding sediment. The decrease of SRR with depth was steeper at Site U1545C than at Site U1546D and follows the respective decrease in sulfate concentration and thus, the position of the SMTZ, which is much shallower at Site U1545C than at Site U1546D (Figures 3E,F). It appears that presently sulfate availability exerts a much stronger control on SRR than past heating of the sediment. Another possible explanation for the deeper SMTZ at Site U1546D would be that due to past heating of the sediment, the remaining organic matter is more recalcitrant than at site U1545C, and therefore SRM are less active so sulfate can penetrate deeper into the sediment. Detailed organic geochemical analyses of the release potential for short-chain organic compounds (Glombitza et al., 2009) could provide insight into this matter.

Heating apparently has a positive effect on microbial activity. At Site U1548C, which has an extremely steep geothermal gradient of 958°C km^−1^ SRR are much higher than at all other sites. We acknowledge that sulfate concentrations during incubations of samples from Site U1545C and Site U1546D were all set to 5 mM and therefore deviate more strongly from porewater concentrations than for samples from other sites, where we set the sulfate concentration individually to approximate *in situ* values (Figures 4G–I). Still, these deviations cannot explain differences in rates over several orders of magnitude between the sites, while still maintaining SRR vs. depth profiles that appear to be closely controlled by porewater sulfate concentration, even when incubation conditions differ considerably from *in situ* conditions.

Site U1548C suggests that heating does have a positive effect on microbial activity. However, the comparison between sites U1545C and U1546D shows that heating caused by sill intrusion at Site U1546D is only temporary. For a more detailed understanding of the influence of sill intrusion and local heating on subsurface microbiology, it would be necessary to recover a suite of sediment samples affected by the entire emplacement and cooling history of sills, plus their non-intruded counterparts for comparison.

### Effect of Temperature on SRR

The drill cores from both sites U1545C and U1546D cover intervals with an *in situ* temperature between *ca*. 40 and 60°C in which all SRR measurements are below MQL (Figures 3C,D). This temperature is close to the shift between mesophiles (15°C–45°C) and thermophiles (45°C–80°C). In non-hydrothermal subseafloor sediment samples from Nankai Trough (IODP Exp. 370), Heuer et al. (2020) also observed a decrease in cell density in sediment around 50°C and an increase of endospores, which they interpreted as a shift in community composition. It appears that the decrease in SRR over the mesophilic to thermophilic temperature transition is not restricted to a specific site or hydrothermal/non-hydrothermal environment. Using slurries of surficial Guaymas Basin sediment, Elsgaard et al. (1994) could quantify SRR between *ca*. 40 and 60°C, but for samples without addition of short-chain fatty acids this temperature interval also represents a local minimum of activity. They also showed that addition of carbon sources not only caused a general increase in SRR, but also a shift of the temperature optimum from almost 70°C to <60°C. In our experiments, we did not add any carbon sources to the incubation and the results (Figures 3C,D) show a similar SRR-temperature profile as the data of Elsgaard et al. (1994) for the non-amended slurries, with a local minimum around 40°C–60°C, followed by an increase and local maximum around 65°C.

At sites U1551B and U1552B sulfate-reducing activity is strictly limited to the psychrophilic temperature range as the respective deepest samples from these sampling sites (7.4 and 16.2°C) reveal SRR that are already below the MQL. At least for Site 1552B, these samples roughly fall into a temperature range that marks the shift between psychrophiles (0°C–20°C) and mesophiles (15°C–45°C). At Site U1551B, the temperature range is especially limited and SRR can only be observed up to 6.6°C. The sediment at this site was more influenced by terrigenous sources than at the other sites drilled in Guaymas Basin during Exp. 385 and the thermal gradient is the lowest among the five sampling sites used in this study (Teske et al., 2020). A more detailed characterization of the organic matter is still lacking, so we can only assume that the sedimentary organic material is already more recalcitrant at the time of deposition, causing a more rapid decrease in substrate availability and hence, microbial activity. Additionally, thermal cracking starts at greater depth due to the comparatively lower geothermal gradient, pushing the potential source of substrates for subsurface life even deeper.

Site U1548C, which has the steepest geothermal gradient of all eight sites drilled during Exp. 385 (958°C km^−1^) forms a stark contrast, in that all measured SRR are above the MQL. SRM are neither limited by electron acceptors, as sulfate concentrations are high throughout the core, nor by electron donors. There is abundant sedimentary organic matter (4.2 wt% of TOC at 2.5 mbsf) and thermal cracking of kerogen due to the onset of catagenesis (60°C) around 60 mbsf might become quantitatively significant in the SMTZ, leading to a supply of both methane and other short-chain organic compounds. However, the temperature at which catagenesis starts and/or becomes quantitatively important depends on the composition of the sedimentary organic matter. As we do not have such information for the IODP Exp. 385 drill cores, we can only assume that the temperature will be roughly in the same range as in Nankai Trough (Horsfield et al., 2006). The ample supply of electron donors is also reflected in the comparatively high SRR throughout the entire core and the lack of a drop in SRR at the mesophilic-thermophilic transition. Elsgaard et al. (1994) also showed in their slurry experiments that carbon addition not only leads to higher SRR, but also less variation in SRR with temperature.

Given the low cell abundances in parts of the cores recovered by Exp. 385 (Morono et al., 2017, pers. comm.) investigations of microbial community structure in these samples is challenging. Thus, our interpretations of changes sulfate reducer community await confirmation by molecular biological analyses.

## Conclusion

We measured SRR in deep subseafloor sediment from several sites in Guaymas Basin which were expected to have high microbial activity and abundance compared to other non-hydrothermal subsurface sediments due to their high content of bioavailable organic matter. Sulfate-reducing activity in Guaymas Basin was detected down to nearly 300 mbsf but at most sites only with low turnover rates of approximately 0.5 pmol cm^−3^ d^−1^. By contrast, SRR near the seafloor are much higher, reaching 10 to 100s of nmol cm^−3^ d^−1^. The highest SRR of 387 nm cm^−3^ d^−1^ was found at Site U1548C, where the sediment is very organic-rich and the geothermal gradient is roughly 1,000°C km^−1^, supposedly leading to thermal cracking of organic matter already at shallow depth. The wide range of the current temperatures in these drill cores requires microbial communities with different temperature optima, ranging from psychrophiles over mesophiles to thermophiles. There is some indication that heating of the sediment leads to elevated microbial activity, but it appears that this effect is only temporarily and ceases once the temperature has decreased again.

## Data Availability Statement

The original contributions presented in the study are included in the article/**Supplementary Material**; further inquiries can be directed to the corresponding author.

## Author Contributions

TN, FS, and JK designed study and analyzed data. FS collected samples. TN carried out the experiments. TN wrote manuscript with input from all coauthors. All authors contributed to the article and approved the submitted version.

## Funding

TN and FS are funded through DFG grants to JK (grant # 670521 and 651694).

## Conflict of Interest

The authors declare that the research was conducted in the absence of any commercial or financial relationships that could be construed as a potential conflict of interest.

## Publisher’s Note

All claims expressed in this article are solely those of the authors and do not necessarily represent those of their affiliated organizations, or those of the publisher, the editors and the reviewers. Any product that may be evaluated in this article, or claim that may be made by its manufacturer, is not guaranteed or endorsed by the publisher.

## Footnotes

^1^ https://rosetta.iodp.tamu.edu/A/TechDoc/5682?encoding=UTF-8

^2^ https://rosetta.iodp.tamu.edu/A/TechDoc/7146?encoding=UTF-8

^3^ http://www-odp.tamu.edu/publications/pubs_tn.htm

^4^ https://web.iodp.tamu.edu/OVERVIEW/

## Supplementary Material

Supplementary Table S1 List of all sulfate reduction rate measurements.
